# Di-(2 ethylhexyl) phthalate and flutamide alter gene expression in the testis of immature male rats

**DOI:** 10.1186/1477-7827-7-104

**Published:** 2009-09-26

**Authors:** Thuy TB Vo, Eui-Man Jung, Vu Hoang Dang, Yeong-Min Yoo, Kyung-Chul Choi, Frank H Yu, Eui-Bae Jeung

**Affiliations:** 1 Laboratory of Veterinary Biochemistry and Molecular Biology, College of Veterinary Medicine, Chungbuk National University, Cheongju, Chungbuk, 361-763, Republic of Korea; 2 School of Dentistry, Seoul National University, Seoul, 110-768, Republic of Korea

## Abstract

We previously demonstrated that the androgenic and anti-androgenic effects of endocrine disruptors (EDs) alter reproductive function and exert distinct effects on developing male reproductive organs. To further investigate these effects, we used an immature rat model to examine the effects of di-(2 ethylhexyl) phthalate (DEHP) and flutamide (Flu) on the male reproductive system. Immature male SD rats were treated daily with DEHP and Flu on postnatal days (PNDs) 21 to 35, in a dose-dependent manner. As results, the weights of the testes, prostate, and seminal vesicle and anogenital distances (AGD) decreased significantly in response to high doses of DEHP or Flu. Testosterone (T) levels significantly decreased in all DEHP- treated groups, whereas luteinizing hormone (LH) plasma levels were not altered by any of the two treatments at PND 36. However, treatment with DEHP or Flu induced histopathological changes in the testes, wherein degeneration and disorders of Leydig cells, germ cells and dilatation of tubular lumen were observed in a dose-dependent manner. Conversely, hyperplasia and denseness of Leydig, Sertoli and germ cells were observed in rats given with high doses of Flu. The results by cDNA microarray analysis indicated that 1,272 genes were up-regulated by more than two-fold, and 1,969 genes were down-regulated in response to DEHP, Flu or both EDs. These genes were selected based on their markedly increased or decreased expression levels. These genes have been also classified on the basis of gene ontology (e.g., steroid hormone biosynthetic process, regulation of transcription, signal transduction, metabolic process, biosynthetic process...). Significant decreases in gene expression were observed in steroidogenic genes (i.e., Star, Cyp11a1 and Hsd3b). In addition, the expression of a common set of target genes, including CaBP1, Vav2, Plcd1, Lhx1 and Isoc1, was altered following exposure to EDs, suggesting that they may be marker genes to screen for the anti-androgenic or androgenic effects of EDs. Overall, our results demonstrated that exposure to DEHP, Flu or both EDs resulted in a alteration of gene expression in the testes of immature male rats. Furthermore, the toxicological effects of these EDs on the male reproductive system resulted from their anti-androgenic effects. Taken together, these results provide a new insight into the molecular mechanisms underlying the detrimental impacts of EDs, in regards to anti-androgenic effects in humans and wildlife.

## Background

Over the past two decades, the detrimental effects of endocrine disruptors (EDs) on wildlife and humans have become a major public health concern. Endocrine disruptors, a large group of environmental pollutants, are believed to act as agonists or antagonists of androgens and estrogens, which are key hormones involved in many physiological processes. These pollutants have been linked to male reproductive defects in humans, including an increase in the incidence of testicular cancer [1], and declining semen quality [2]. Evidence of cryptorchidism, undescended testis and hypospadias have also been demonstrated [3]. In addition, EDs have been linked to developmental problems in the testis and reproductive tract, including reductions in fertility and litter size, induction of cryptorchidism and testicular atrophy [4,5].

Normal development of the male reproductive tract requires interactions between many biological factors and hormones. In particular, androgen hormones are essential to this process. However, many environmental chemicals have androgenic or anti-androgenic effects, or can mimic androgenic activities (i.e., thereby stimulating an androgen-dependent response). Adverse trends in human and animal male reproductive health, particularly with regards to the regulation of environmental factors, suggest that future generations will be at greater risk. Previous reports have suggested that male reproductive system disorders, which often originate during the fetal stage, can appear as testicular dysgenesis syndrome (TDS) after birth [6].

Previous studies demonstrated the possible effects of antiandrogenic- EDs [i.e., flutamide and/or di- (2 ethylhexyl) phthalate] on the reduction of androgen synthesis during the development of the male reproductive tract [7,8]. These EDs appear to induce abnormalities in the formation of external genitalia, i.e., hypospadias, cryptorchidism and agenesis of the epididymis, vas deferens and prostate. In additional, the effects of these EDs were also observed with regards to AGD and nipple retention [5]. In humans, some of these alterations are permanent and affect testes function later in life [9]. Although ED-induced harmful effects on male reproduction have been demonstrated, the molecular mechanisms by which EDs disrupt testis development and affect testicular dysgenesis are not clearly understood.

Di-ethylhexyl phthalate (DEHP) is widely used as a plasticizer in commercial products [10]. The effects of DEHP on male reproductive development have been well studied in rats [11]. In addition, phthalates and their metabolites can be released from such products and have been detected in the environment [12], posing potential health risks for humans and wildlife. Infants may be exposed to phthalates in the womb [13], via breastfeeding [14] or from medical devices in neonatal intensive care units [15]. Although DEHP has been reported to modulate fetal testosterone production [16], testicular physiology, and mammalian reproduction and fertility [17], the exact mechanisms by which DEHP exerts detrimental effects on body have not yet been fully elucidated. Previous studies have demonstrated the adverse effects of DEHP on the hypothalamic-pituitary-gonadal axis in neonatal female rats, as well as on ex vivo steroidogenesis in granulosa cells (GCs) and secretion of LH by gonadotropes [18]. Moreover, exposure to phthalates during reproductive tract development reduces the number of Sertoli cells (i.e., the major somatic cell type, which supports spermatogenesis) [19]. In addition, DEHP and its metabolites decrease testicular testosterone levels in rodents [20], suggesting potential impacts of these contaminants on Leydig cells. A recent study has indicated that a variety of steroidogenesis related genes were altered following phthalate- exposure and a down-regulation of most of genes involved in testosterone (T) biosynthetic pathways was observed, indicating the potential mechanism for decreased T synthesis induced by phthalate exposure [21]. Other study has reported a similar genetic response in the fetal and prepubertal testes of rats exposed to these environmental chemicals [22].

Flutamide (Flu) is a well-known AR antagonist that is widely used in therapies for androgen-dependent prostate cancer [23] Pre- and postnatal exposure of rats to Flu alters androgen-dependent reproductive development and function [24]. Flu exposure also increases plasma LH levels and stimulates intracellular steroidogenesis in rat testes. In addition, lower ventral prostate and seminal vesicle weights have also been reported, suggesting that Flu exerts anti-androgenic effects on androgen-targeting organs [25]. It has been indicated that exposure of rats to Flu caused a dysregulation in expression of hypothalamus/pituitary hormone genes and consequently this may affect gonadotropin release and induce an over-expression of testicular steroidogenic enzyme genes [26].

In this study, we used an immature rat model of development to examine the adverse effects of DEHP and Flu on the male reproductive system. In particular, we examined the effects of DEHP and Flu on the male reproductive tract and the production of testosterone and LH. We also assessed the histopathological changes of the testis in response to ED exposure. AGD values were used as androgen-dependent markers to assess the anti-androgenic and androgenic activities of the EDs. In addition, we examined alterations in gene expression in the testis of immature rats following DEHP or Flu treatment. Testosterone propionate (TP), an androgen agonist, was used as an indication of androgenic activity in androgen-responsive organs. Our findings will contribute to a better understanding of the detrimental effects of anti-androgenic EDs on humans and wildlife.

## Methods

### Chemicals

Testosterone propionate (TP; # 203-08433) and di-(2-ethlhexyl) phthalate (DEHP; # 80032) were purchased from the Wako Chemical Company (Osaka, Japan). Flutamide (Flu; # F-9397) and corn oil (i.e., used as a vehicle) were obtained from Sigma-Aldrich Ltd. (St Louis, MO, USA).

### Animals and treatment

Sprague-Dawley (SD; 32 males) immature rats were purchased from SamTaKo-Bio Korea (Chungbuk, Korea). The rats were housed in polycarbonate cages in a controlled environment, with an illumination schedule of 12 hour light/12 hour dark. Rats were fed a diet of soy-free pellets (Samyang Ltd., Korea), and water was provided *ad libitum*. All experimental procedures, including those involving animals, were approved by the ethics committee of Chungbuk National University. From postnatal days (PNDs) 21 to 35, rats were treated daily with TP (1 mg/kg body weight [BW]/day), DEHP (10, 100 or 500 mg/kg BW/day), Flu (1, 10 or 50 mg/kg BW/day) via oral gavage or with corn oil (5 ml/kg BW/day) as a vehicle. Dosages were adjusted according to changes in body weight. Body weights, clinical signs and abnormal behaviors were recorded daily throughout the experimental periods. All animals were euthanized by exposure to ethyl ether 24 hours after the final treatment. Blood was collected from the descending vena cava and serum was prepared for hormonal analysis. Changes in the weights of testes, epididymides, prostate and seminal vesicles were recorded. Four testes were collected from each group for total RNA isolation. Other testes were fixed in Bouin's solution, paraffin embedded, and sectioned at 5 μm for histopathological examination.

### Hormonal measurements

After collecting blood from the abdominal aorta, serum was prepared and stored at -20°C for testosterone and LH analyses. The Testosterone Enzyme Immunoassay kit (No. 900-065) was obtained from Assay Designs, Inc (Ann Arbor, USA) and the LH Detect Kit was purchased from INRA (Nouzilly, France). ELISA tests were performed according to the manufacturer's recommendations. The coefficients of variation (CV) of intra- and inter-assays were 4% and 14%.

### Total RNA preparation

Total RNA was extracted from the testes of immature male rats using Trizol (Invitrogen, Carlsbad, CA, USA), according to the manufacturer's recommendations. To make cDNAs from mRNAs for microarray analysis, the same quantity of each RNA sample from the treated groups or control group was pooled. The concentration and quality of each RNA sample was determined by measuring absorbances at 260 nm and 280 nm.

### cDNA microarray analysis of testes

The oligo chip of GeneChip^® ^Rat Genome 230 2.0 Array (Affymetrix, CA, USA), containing over 31,000 probe sets and representing over 28,000 well-substantiated rat genes, were used according to the supplier's instructions. Briefly, total RNA (i.e., 30 μg) from each testis was converted to cDNA for use for microarray analyses. The cDNA products were subsequently subjected to *in vitro *transcription using biotinylated cytidine 5'-triphosphate and uridine-5'-triphosphate. Fluorescent labels were incorporated during reverse-transcription of pooled poly (A)^+ ^RNA from the testes of animals in the DEHP, Flu or control groups, after priming with oligo (dT) primers (Ambion, Austin, TX). Four replicates per group were used for hybridization, thus all together 12 microarrays (3 groups × 4 biological replicates) were employed to evaluate gene alteration by EDs in this study. The fluorescent targets (i.e., Cy3-dCTP controls and Cy5-dCTP experimental cDNAs) were mixed, added to the microarray surface and covered with a cover slip. After overnight incubation at 65°C in a humidified environment, microchips were washed and scanned using a GeneChip Scanner 3000 (Affymatrix, Inc, CA, USA). Quantitative values for signals were calculated using GenePix Pro software, version 5.1 (Molecular Devices Co., CA, USA). Data analysis was performed using the GenePlex (Istech, Inc., Korea). Logged gene expression ratios were normalized by LOWESS (Locally weighted scatter plot smoother) regression as previously described [27]. The biological pathways of the genes were classified based on pathway analysis in which the classification of pathway of interesting genes was determined based on Database for Annotation Visualization and Integrated Discovery (DAVID) .

The statistical significance of microarray gene expression was assessed by computing a q-value for each gene. To determine the q-value, we used a permutation procedure, and for each permutation a two-sample *t*-statistic was computed for each gene. The result was considered significant when the logarithmic gene expression ratio of four independent hybridizations was more than twofold the difference in the expression level. The accuracy of microarray analysis in this study was confirmed by real-time PCR as previously done [28].

### Real-time PCR analysis

The results of cDNA microarray analyses were confirmed and validated via real-time PCR. The primers sequences used in real-time PCR analysis are described in Table 1. Samples were analyzed in a 20 μl reaction volume containing 10 μl of SYBR premix Ex Taq (TaKaRa Bio., Inc.) using a 7300 Real-Time PCR system (Applied Biosystems, Foster, CA, USA), following the manufacturer's recommendations. The relative expression level of each gene was normalized to that of HPRT (i.e., an internal control gene) and quantified using RQ software (Applied Biosystems).

**Table 1:** Primer sequences for Real-time PCR analyses of gene expression

**Transcript ID**	**Gene Symbol**		**Sequense (5'-3')**	**Size**
NM_031558	*StAR*	Forward	tcaaggaatcaaggtcctg	208
		Reverse	tgttcagctctgatgacacc	
NM_017286	*Cyp11a1*	Forward	Atccagcttctttcccaatc	229
		Reverse	caggatgaggttgaacttgg	
NM_017265	*HSD3b*	Forward	Cgctgctgtcattgatgtct	299
		Reverse	tatgcagtgtgccaccattt	
NM_133529	*Cabp1*	Forward	tgactttgtggaactgatgg	232
		Reverse	gaagtccactcgtccatctc	
XM_216030	*Vav2*	Forward	cagaggagacggctgaaaac	338
		Reverse	gatgaggtcctccaggttga	
NM_145880	*Lhx1*	Forward	Ttctggaccgtttcctcttg	198
		Reverse	gaaccagatcgcttggagag	
NM_001014242	*Isocl*	Forward	acacgtctgtatccagcaga	227
		Reverse	Tggccttaattaggttctgg	
NM_017035	*Plcd1*	Forward	agctgccaaaggtcaataag	238
		Reverse	ctctggccaataaagtcgtt	

### Histopathological examination of testis

Testis tissues were fixed in Bouin's solution and immersed in neutral formalin solution. The fixed tissues were embedded in paraffin, sectioned at 5 μm and mounted on slides. These sections were stained with hematoxylin and eosin (H&E), and histopathological changes were examined under a light microscope.

### Statistical analyses

Results are presented as means ± standard deviations (SD). Absolute body weights, sexual organ weights and AGD measurements were analyzed at the time of necropsy. When significant changes were detected, Tukey's multiple regression test was used to compare the treatments (i.e., by comparing the control and experimental groups). Data were considered statistically significant at *p *< 0.05.

## Results

### Effects of DEHP and Flu on body weight, reproductive organ weight and anogenital distances

**Figure 1:**
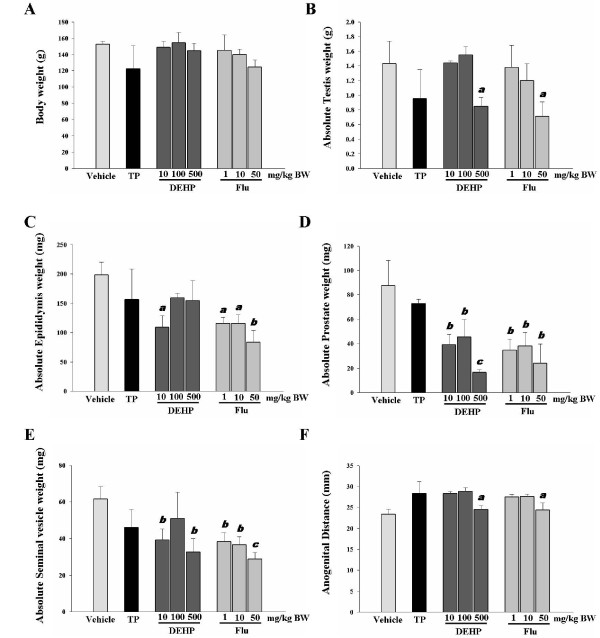
**Effects of TP, DEHP and Flu on body weight, reproductive organ weight, and anogenital distance in immature male rats (n = 4/group)**. The weights of testes, epididymis, prostate and seminal vesicles were recorded. Data were analyzed by ANOVA, followed by Tukey's multiple regression. a: p < 0.05; b: p < 0.01 and c: p < 0.001, compared with a control.

Exposure of immature male rats to DEHP or Flu did not cause any significant changes in body weight (Figure 1A). However, a high doses of DEHP (i.e., 500 mg/kg BW/day) or Flu (i.e., 50 mg/kg BW/day) significantly decreased the weights of reproductive organs (e.g. testis, prostate and seminal vehicle weights) as shown in Figure 1. Interestingly, the diminution of epididymis weight was detected in a smaller dose of DEHP and all doses of Flu treatment group. As expected, the high doses of DEHP and Flu significantly decreased AGD when compared with a vehicle, demonstrating the potential effects of DEHP or Flu on androgen-responsive organs (Figure 1F). However, any significant effect in the androgen- dependent organs in animals treated with TP was not observed even though there is a tendency of gene alterations by TP as seen Figure 1. These results suggest that androgens have been linked with alterations in several end points measured in the immature male onset assay, but seem likely that higher doses of TP may cause this.

### Effects of DEHP and Flu on serum concentrations of testosterone and LH

**Figure 2:**
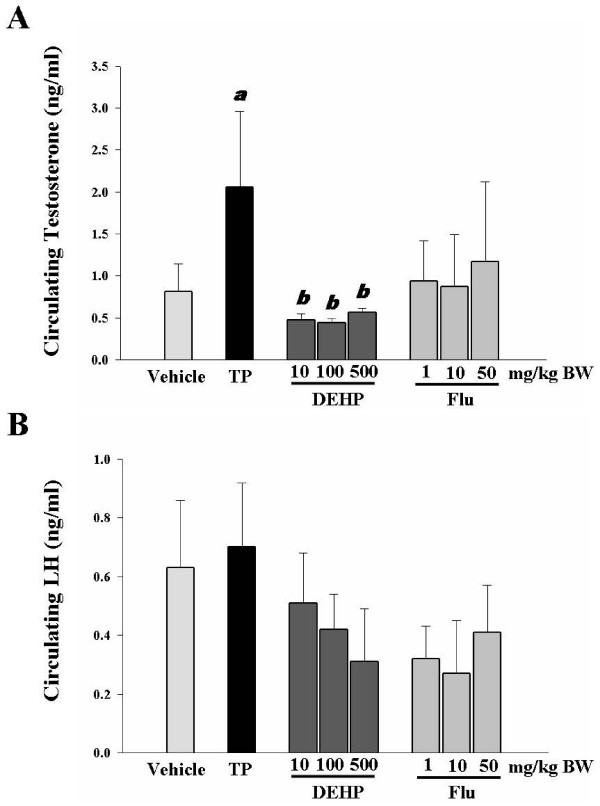
**Effects of TP, DEHP and Flu on circulating testosterone and LH levels in immature male rats**. **A**. Serum testosterone concentration. **B**. Serum LH concentrations were altered by ED exposure (i.e., n = 4). Data are shown as mean ± SEM. Significant differences were noted relative to a control (i.e., a, p < 0.05) or TP (i.e., b, p < 0.05).

To further explore the effects of DEHP and Flu on critical stages in the development of the male reproductive system, serum concentrations of testosterone and LH were measured using an ELISA kit. As shown in Figure 2, a significant decrease in the levels of testosterone was observed when rats were exposed to all doses of DEHP (i.e., 10, 100, 500 mg/kg BW/day). However, no significant alteration by any of the two treatments was observed in the serum levels of LH, although these compounds showed a tendency to decrease LH levels. TP treatment as an androgen agonist increased the circulating testosterone without affecting the levels of LH. These data suggest that DEHP exerts an effect on the circulating levels of testosterone in immature male rats.

### Effects of DEHP and Flu on histopathology of the testes

**Figure 3:**
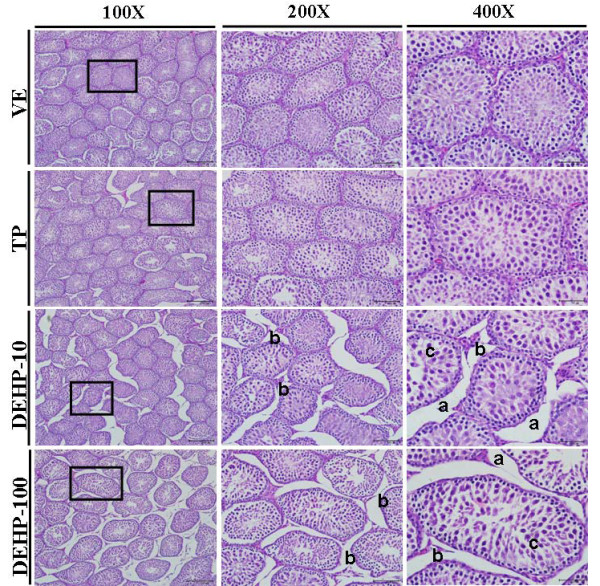
**Effects of TP, DEHP (10, 100 mg/kg BW/day) on histopathological changes in immature male rats exposed to EDs from PND 21 to PND 35**. Testis tissues were fixed in Bouin's solution and immersed in neutral formalin solution. The fixed tissues were embedded in paraffin, sectioned at 5 μm and mounted on slides. These sections were stained with hematoxylin and eosin (i.e., HE) and histopathological changes were assessed under a light microscope. Dilatation of the tubular lumen (**a**: stained signals), degeneration of Leydig cells (**b**: stained signals), and disorder of germ cells (**c**: stained signals) were observed in the testes of immature male rats. Results are shown at a 100 × magnification (i.e., bar = 200 μm), a 200 × magnification (i.e., bar = 100 μm) and a 400 × magnification (i.e., bar = 50 μm).

**Figure 4:**
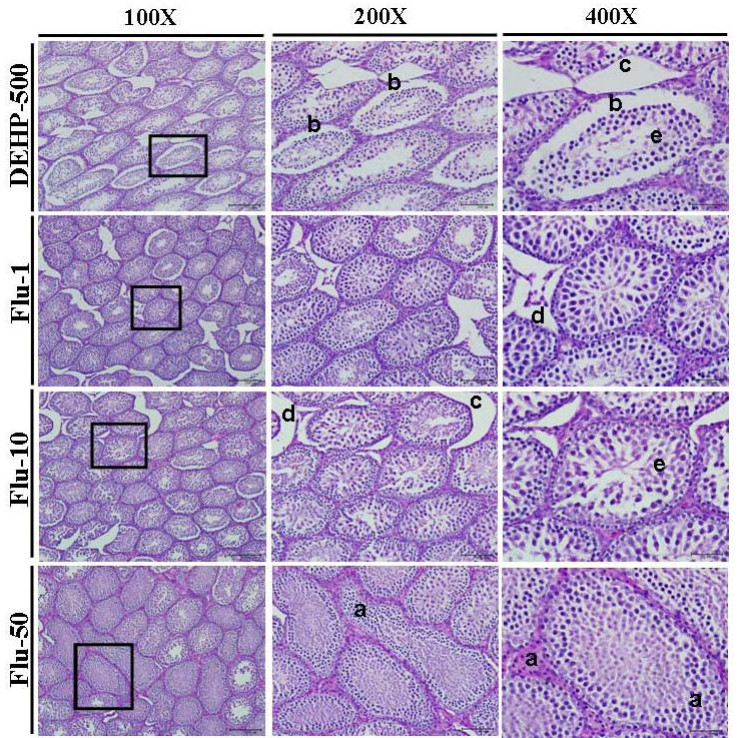
**Effects of DEHP (500 mg/kg BW/day) and Flu on histopathological changes in immature male rats exposed to EDs from PND 21 to PND 35**. Testis tissues were fixed in Bouin's solution and immersed in neutral formalin solution. The fixed tissues were embedded in paraffin, sectioned at 5 μm and mounted on slides. These sections were stained with hematoxylin and eosin (i.e., HE) and histopathological changes were assessed under a light microscope. Hyperplasia of Leydig cells, germ cell (**a**: stained signals), stratification of germ cells (**b**: stained signals), dilatation of the tubular lumen and stratification (**c**: stained signals), degeneration of Leydig cells (**d**: stained signals), and disorder of germ cells (**e**: stained signals) were observed in the testes of immature male rats. Results are shown at a 100 × magnification (i.e., bar = 200 μm), a 200 × magnification (i.e., bar = 100 μm) and a 400 × magnification (i.e., bar = 50 μm).

Histopathological abnormalities in the testes of immature male rats exposed to DEHP and Flu were examined by hematoxylin and eosin (H&E) staining, as described in the *Materials and Methods*. Testes are androgen-responsive tissues; thus, alterations in the morphology and histology of testes can result from the anti-androgenic effects of chemicals. As seen in Figure 3 and 4, testes morphology and histology were influenced by treatment with DEHP and Flu, respectively. Degeneration of Leydig cells and disorders of germ cells in the reproductive tract were noted in response to all doses of DEHP and Flu. In addition, dilatation of the tubular lumen and stratification of germ cells were observed when rats were treated with DEHP (i.e., 100 and 500 mg/kg BW/day) (Figure 3 and 4). Hyperplasia of Leydig and Sertoli cells appeared in rat testes at PND 36 in response to high doses of Flu (i.e., 50 mg/kg BW/day) (Figure 4). In addition, widespread germ cells disorder or leydig cells degeneration resulted from treatment with all dose of DEHP and Flu (Figure 3 and 4). These results demonstrate the adverse effects of anti-androgenic EDs on the male reproductive tract, particularly with regards to spermatogenesis.

### Effects of DEHP and Flu on gene expression in the testes

**Figure 5:**
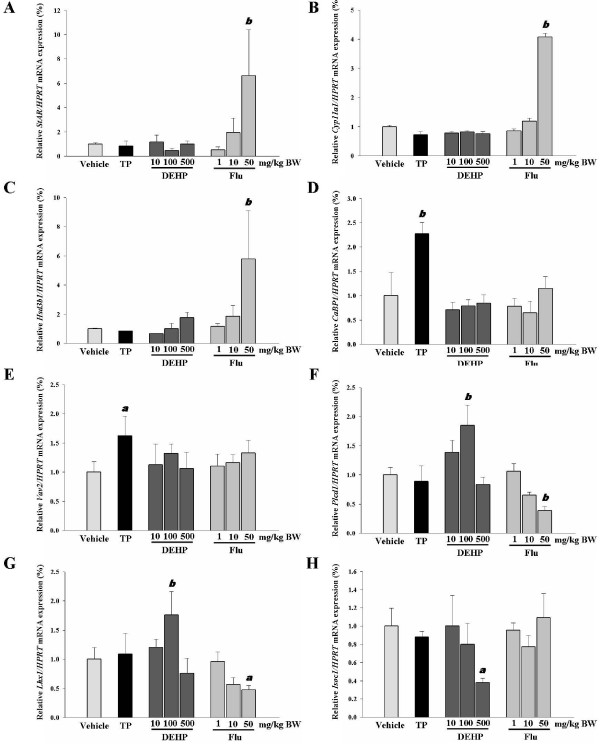
**Altered gene expressions in steroidogenesis-related genes (i.e., *StAR, Cyp11a1, HSD3b1*), and a common gene set containing known TP, DEHP or Flu markers (i.e., *CaBP1, Vav2, Plcd1, Lhx1, Isoc1*)**. Altered gene expressions were expressed relative to controls by real-time PCR as described in the Materials and Methods. Data are presented as means and SEMs (i.e., n = 4 for each group). Asterisks denote significant differences, relative to a control (i.e., p < 0.05).

Gene expression in immature rat testes in response to TP, DEHP or Flu was assessed using cDNA microarrays. In total, 37,317 of 41,016 genes were dysregulated following treatment with DEHP (i.e., 100 mg) or Flu (i.e., 10 mg), when compared with a control. Among these genes, 1,272 genes were up-regulated over 2-fold change and 1,969 genes were downregulated by more than two-fold. Specific genes were then selected as marker genes for androgenic or anti-androgenic activities of TP, DEHP, or Flu (Table 2 and 3), respectively, including *Orc4l*, *Mgat4a*, and predicted *Scrt1*, *Tmem93*, *RGD1308066*, *Omp, RGD1561053, Cnot3, Ubxd6, RGD1561121, Olr297 *and *Cilp *genes. Altered genes have been also classified on the basis of gene ontology (e.g., steroid hormone biosynthetic process, regulation of transcription, signal transduction, metabolic process, catabolic process, biosynthetic process, and integral to membrane, mitochondrion and others) as shown in Table 4. However, the biological interactions of these proteins in response to EDs (i.e., particularly DEHP and Flu) remains unknown. Six genes (i.e., *CaBP1, Vav2, Plcd1, Lhx1, Hsd3b *and *Isoc1*) were randomly selected to validate the cDNA microarray results via real-time PCR (Figure 5). The results of these analyses suggest that these genes may be useful markers to screen for anti-androgenic effects of EDs in androgen-responsive tissues. In addition, two well-known marker genes (i.e., *StAR *and *Cyp11a1*) were used to screen for the anti-androgenic effects of these EDs. As expected, the expressions of steroidogenesis-related genes (i.e., *StAR *and *Cyp11a1*) were significantly affected by Flu exposure (i.e., 50 mg/kg BW/day) (Figures 5A and 5B). To confirm the correlation between microarray data and real-time PCR, we plotted the fold changes (*versus *vehicle) of microarray and real-time PCR results. There was a good correlation between the results obtained from microarray and real-time PCR (R^2 ^= 0.824722106, *p *< 0.0001). Taken together, these results suggest that exposure to DEHP and Flu resulted in an alteration of gene expression in the testis of immature male rats. The distinct transcriptional response to DEHP and Flu reflect that these EDs may exert their anti-androgenic effects via different mechanisms.

**Table 2:** Up-regulated gene induced by TP, DEHP and Flu in the immature rat testes.

**Transcript ID**	**Gene Symbol**	**Gene Name**	**Fold change (compared with VE)**		
			**vs TP**	**vs DEHP 100**	**vs Flu 10**
**TP UP**					
XM_345848	*Scrt1_predicted*	scratch homolog 1, zinc finger protein (Drosophila)	**5.88**	1.84	1.46
XM_213394	*Tmem93_predicted*	transmembrane protein 93 (predicted)	**5.07**	1.49	1.43
XM_215951	*Kcng1*	potassium voltage-gated channel, subfamily G, member 1	**3.07**	1.13	0.96
NM_001008295	*Fip1l1*	FIP1 like 1 (S. cerevisiae)	**2.44**	1.00	1.08
NM_021669	*Ghrl*	ghrelin precursor	**2.37**	1.02	1.00
XM_221387	*Etv5_predicted*	ets variant gene 5 (ets-related molecule) (predicted)	**2.14**	1.01	1.13
XM_215635	*Apoa1bp_predicted*	apolipoprotein A-I binding protein (predicted)	**2.11**	1.31	1.22
NM_031802	*Gabbr2*	gamma-aminobutyric acid (GABA) B receptor 2	**2.06**	1.29	0.80
NM_133529	Cabp1*	calcium binding protein 1	**2.05**	1.11	0.97
XM_216030	Vav2*	Vav2 oncogene (predicted)	**1.77**	1.41	1.11
**DEHP UP**					
XM_213289	*RGD1308066_predicted*	similar to KIAA1960 protein (predicted)	0.99	**3.29**	1.64
NM_012616	*Omp*	olfactory marker protein	1.07	**3.08**	1.19
NM_021590	*Aipl1*	aryl hydrocarbon receptor-interacting protein-like 1	1.31	**2.91**	1.22
NM_031770	*Gnb5*	guanine nucleotide binding protein, beta 5	1.14	**2.65**	1.10
NM_001000980	*Olr1366*	olfactory receptor 1366	1.33	**2.62**	1.16
NM_022625	*Tpc1808*	tropic 1808	1.11	**2.43**	1.36
NM_001014015	*Rbm34*	RNA binding motif protein 34	1.28	**2.43**	1.22
NM_199291	*Doxl2*	diamine oxidase-like protein 2	1.27	**2.42**	1.01
XM_220283	*Sox8_predicted*	SRY-box containing gene 8 (predicted)	1.10	**2.33**	1.18
NM_023992	*Kiss1r*	KISS1 receptor	1.12	**2.31**	1.24
NM_001000365	*Olr748_predicted*	olfactory receptor 748 (predicted)	1.21	**2.30**	1.26
NM_017035	Plcd1*	phospholipase C, delta 1	1.20	**2.20**	1.24
XM_221627	*Brwd1_predicted*	bromodomain and WD repeat domain containing 1 (predicted)	1.30	**2.19**	1.18
XM_221521	*Hoxd4_predicted*	homeo box D4 (predicted)	0.67	**2.17**	0.93
XM_344627	*Calm4_predicted*	calmodulin 4 (predicted)	1.08	**2.16**	0.84
XM_216225	*Ppp4r2_predicted*	protein phosphatase 4, regulatory subunit 2 (predicted)	1.22	**2.15**	1.45
NM_175587	*Taar7h*	trace-amine-associated receptor 7 h	1.14	**2.14**	1.11
NM_020106	*Olr414_predicted*	olfactory receptor 414 (predicted)	1.35	**2.13**	0.94
NM_145880	Lhx1*	LIM homeobox protein 1	1.04	**1.72**	1.20
**Flu UP**					
XM_575355	*RGD1561053_predicted*	similar to Claudin 12 (predicted)	1.49	1.16	**3.18**
XM_218187	*Cnot3_predicted*	CCR4-NOT transcription complex, subunit 3 (predicted)	1.21	1.10	**2.48**
NM_012992	*Npm1*	nucleophosmin 1	1.31	1.13	**2.25**
NM_017265	Hsd3b1*	hydroxy-delta-5-steroid dehydrogenase, 3 beta- and steroid delta-isomerase 1	0.87	0.97	**2.18**
XM_214673	*Cog8_predicted*	component of oligomeric golgi complex 8 (predicted)	0.75	0.79	**2.07**
XM_225336	*Lrrc16_predicted*	leucine rich repeat containing 16 (predicted)	1.00	1.31	**2.06**
XM_226843	*Rnasen*	ribonuclease III, nuclear	0.93	0.69	**2.05**
XM_234056	*RGD1564242_predicted*	similar to KIAA1218 protein (predicted)	0.97	1.02	**2.05**
NM_013175	*Sgne1*	secretory granule neuroendocrine protein 1	0.83	0.81	**2.04**
XM_221047	***Polg2_predicted***	polymerase (DNA directed), gamma 2, accessory subunit (predicted)	1.14	1.05	**2.01**

The cDNA microarray analysis for detection of fold changes of gene expression in the testis of immature rats following treatments with TP, DEHP or Flu.

Altered gene up-regulation expression was estimated by the ratio of TP, DEHP 100 mg/kg BW/day and Flu 10 mg/kg BW/day treated vs. control as follows.

*, These altered genes were selected for verification by real- time PCR.

**Table 3:** Down-regulated gene induced by TP, DEHP and Flu in the immature rat testes.

**Transcript ID**	**Gene Symbol**	**Gene Name**	**Fold change (compared with VE)**		
			**vs TP**	**vs DEHP 100**	**vs Flu 10**
**TP DOWN**					
NM_001012225	*Mgat4a*	Mannoside acetylglucosaminyltransferase 4, isoenzyme A	**0.30**	0.69	0.74
XM_214360	*Ubxd6_predicted*	UBX domain containing 6 (predicted)	**0.35**	0.82	0.63
XM_342534	*Nat5_predicted*	N-acetyltransferase 5 (ARD1 homolog, S. cerevisiae) (predicted)	**0.39**	0.77	1.03
NM_053356	*Col1a2*	procollagen, type I, alpha 2	**0.42**	0.81	0.79
XM_345107	*RGD1559810_predicted*	similar to hypothetical protein (predicted)	**0.42**	0.83	0.77
NM_133317	*Tob1*	transducer of ErbB-2.1	**0.43**	0.82	1.04
XM_341903	*Nrip3_predicted*	nuclear receptor interacting protein 3 (predicted)	**0.44**	1.90	1.87
XM_229139	*RGD1566225_predicted*	similar to RIKEN cDNA 1700001F22 (predicted)	**0.44**	1.00	0.82
NM_001013185	*Cabc1*	chaperone, ABC1 activity of bc1 complex like (S. pombe)	**0.45**	1.08	0.73
XM_235640	*Tmem16f_predicted*	transmembrane protein 16F (predicted)	**0.46**	0.87	0.68
NM_001010966	*Pigv*	phosphatidylinositol glycan, class V	**0.49**	2.21	1.73
NM_133318	*Khdrbs2*	KH domain containing, RNA binding, signal transduction associated 2	**0.49**	0.82	0.85
**DEHP DOWN**					
XM_218447	*RGD1561121_predicted*	similar to pleckstrin homology-like domain, family B, member 3 (predicted)	1.26	**0.12**	0.99
NM_199092	*Orc4l*	origin recognition complex, subunit 4-like (S. cerevisiae)	0.70	**0.40**	0.72
NM_031753	*Alcam*	activated leukocyte cell adhesion molecule	0.75	**0.46**	0.80
NM_001014242	Isoc1*	isochorismatase domain containing 1	0.85	**0.46**	0.90
XM_215497	*Rad1_predicted*	RAD1 homolog (S. pombe) (predicted)	0.97	**0.46**	0.86
XM_575065	*RGD1559623_predicted*	similar to RIKEN cDNA 5230400J09 (predicted)	0.93	**0.48**	0.69
NM_139189	*Lmbrd1*	LMBR1 domain containing 1	0.76	**0.48**	0.74
XM_236578	*RGD1562949_predicted*	similar to mKIAA0259 protein (predicted)	0.91	**0.49**	1.26
XM_215487	*Mrps30_predicted*	mitochondrial ribosomal protein S30 (predicted)	0.90	**0.49**	0.76
**Flu DOWN**					
NM_001000234	*Olr297_predicted*	olfactory receptor 297 (predicted)	1.02	1.54	**0.25**
XM_236348	*Cilp_predicted*	cartilage intermediate layer protein, nucleotide pyrophosphohydrolase (predicted)	0.65	0.89	**0.40**
XM_228462	*RGD1565441_predicted*	similar to KIAA1687 protein (predicted)	0.90	1.02	**0.43**
XM_219296	*Rbbp6*	retinoblastoma binding protein 6	0.65	0.82	**0.45**
NM_021653	*Dio1*	deiodinase, iodothyronine, type I	0.97	0.97	**0.46**

Altered gene down-regulation expression was estimated by the ratio of TP, DEHP 100 mg/kg BW/day and Flu 10 mg/kg BW/day treated vs. control as follows.

*, These altered genes were selected for verification by real- time PCR.

**Table 4:** Functional Categorization of Genes Significantly Altered via microarray analysis in immature male testes following TP, DEHP and Flu exposure

**Functional category**	**Transcript ID**	**Gene Symbol**
**Steroid hormone biosynthetic process**	NM_031558	*Star*
	NM_017286	*Cyp11a1*

**Regulation of Transcription**	XM_221387	*Etv5_predicted*
	XM_220283	*Sox8_predicted*
	XM_221521	*Hoxd4_predicted*
	NM_145880	*Lhx1*
	XM_218187	*Cnot3_predicted*
	XM_229139	*RGD1566225_predicted*
	XM_221627	*Brwd1_predicted*
	NM_133317	*Tob1*

**Signal Transduction**	NM_031802	*Gabbr2*
	XM_216030	*Vav2_predicted*
	NM_012616	*Omp*
	NM_031770	*Gnb5*
	NM_001000980	*Olr1366*
	NM_023992	*Kiss1r*
	NM_175587	*Taar7h*
	NM_020106	*Olr414_predicted*
	NM_012992	*Npm1*
	NM_031753	*Alcam*
	XM_344627	*Calm4_predicted*

**Metabolic process**	NM_021590	*Aipl1*
	NM_001012225	*Mgat4a*
	XM_342534	*Nat5_predicted*
	NM_001014242	*Isoc1*
	XM_236348	*Cilp_predicted*

**Catabolic process**	NM_022625	*Tpc1808*
	NM_017035	*Plcd1*

**Biosynthetic process**	NM_017265	*Hsd3b1*
	NM_001010966	*Pigv*
	NM_021653	*Dio1*

**Integral to membrane**	XM_213394	*Tmem93_predicted*
	NM_001000365	*Olr748_predicted*
	XM_575355	*RGD1561053_predicted*
	XM_214673	*Cog8_predicted*
	XM_214360	*Ubxd6_predicted*
	NM_001000234	*Olr297_predicted*
	NM_139189	*Lmbrd1*
	XM_235640	*Tmem16f_predicted*

**Mitochondrion**	NM_001013185	*Cabc1*
	XM_215487	*Mrps30_predicted*

**Protein binding**	XM_215635	*Apoa1bp_predicted*
	NM_133529	*Cabp1*
	NM_013175	*Sgne1*
	XM_221047	*Polg2_predicted*
	NM_053356	*Col1a2*
	NM_133318	*Khdrbs2*
	XM_228462	*RGD1565441_predicted*
	XM_219296	*Rbbp6*

**Others**	XM_215951	*Kcng1*
	NM_001008295	*Fip1l1*
	NM_021669	*Ghrl*
	XM_213289	*RGD1308066_predicted*
	NM_022625	*Tpc1808*
	NM_001014015	*Rbm34*
	NM_199291	*Doxl2*
	XM_216225	*Ppp4r2_predicted*
	XM_225336	*Lrrc16_predicted*
	XM_226843	*Rnasen*
	XM_234056	*RGD1564242_predicted*
	XM_345107	*RGD1559810_predicted*
	XM_341903	*Nrip3_predicted*
	XM_218447	*RGD1561121_predicted*
	NM_199092	*Orc4l*
	XM_215497	*Rad1_predicted*
	XM_575065	*RGD1559623_predicted*

## Discussion

There are a number of mechanisms by which EDs may alter the action of the endocrine system and interfere with the reproduction of humans and animals. Previous studies have shown that EDs mimic the actions of natural hormones and can stimulate or inhibit various enzymes required for hormone synthesis. Consequently, EDs interfere with the regulation of gene expression, disrupting the natural hormone balance and interfering with the normal action of the reproductive system. In particular, EDs may exert distinct androgenic and anti-androgenic effects on the developing male reproductive system in utero [29]. Although previous studies have examined the effects of these chemicals on critical stages in the development of the male reproductive system, the relationship between molecular events and detrimental effects of these EDs is not well described. To further explore these effects, we used an immature rat model of development to examine the adverse effects of DEHP and Flu on the male reproductive system. Oral treatments with DEHP (i.e., 10, 100 and 500 mg/kg BW/day) and Flu (i.e., 1, 10 and 50 mg/kg BW/day) failed to induce significant effects on body weight in a dose-dependent manner. However, high doses of DEHP and Flu significantly decreased the weights of reproductive organs, with the exception of the epididymis. Although treatment with high doses of DEHP failed to induce anti-androgenic effects on epididymis weight, these effects were observed after treatment with low doses of DEHP (i.e., 10 mg/kg BW/day). These results provide evidence of the anti-androgenic effects of DEHP in the epididymis. A previous study reported that treatment with DEHP (i.e., 500 mg/kg BW/day) failed to induce significant effects on body weight [30]. However, other studies have shown significant decreases in body weight in response to Flu (i.e., 100 or 150 mg/kg BW/day) [31], suggesting that these EDs differ markedly with regards to their detrimental affects on human and animals. Further studies are required to elucidate the molecular and biochemical mechanisms underlying the effects of anti-androgenic ED exposure in humans and animals, particularly during the critical stages of male reproductive development.

It has been suggested that alterations in endogenous androgen levels or dysregulation of gene expression patterns causes abnormalities in male reproductive tract (i.e., cyptochism, hypospadia, stunted testicular growth, epididymal abnormalities, and AGD [32-34]. In addition, the anti-androgenic and androgenic effects of EDs can be reflected by changes in the weights of the testes, prostate and seminal vesicle. A previous study of rat offspring indicated dose-dependent reductions in the ventral and dorsolateral prostate weights in response to DEHP [35]. Another study reported malformation of the male reproductive tract following exposure to DEHP, manifesting as disgenesis of the epididymis, decreased sperm production, Leydig cell hyperplasia and adenomas [36]. A significant decrease in the weights of rat testes in response to DEHP (i.e., 250, 500, 1000 or 2000 mg/kg BW/day by gavage) has also been reported [37]. Another study demonstrated that the epididymal, prostate and seminal vesicle weights of immature male rats were strongly affected by the anti-androgenic effects of Flu at PND 20 [38]. Previous studies have proposed an androgen-dependent marker for the anti-androgenic and androgenic effects of EDs using AGD. A significant reduction in AGD is evoked by DEHP exposure during lactation [39] and by prenatal exposure to Flu [40]. Moreover, changes in AGD during early postnatal periods correlate with alterations in androgen-dependent development in adults [41]. In the present study, AGD values decreased significantly in response to high doses of DEHP or Flu; however, no alterations were observed after treatment with their medium and low doses. Although there is a correlation between EDs and reproductive, developmental and behavioral changes at high doses in experimental animals, further studies are required to determine if low doses may also contribute to reproductive disorders in humans and wildlife [39].

A recent study has indicated the property of phthalates associated with endocrine disruption and these compounds are believed to act as endocrine disruptors [42]. Exposure to phthalates reduces testosterone synthesis during this critical stage of development [7]. Phthalates exert their effects on steroidogenesis in Leydig cells via modulation of testosterone-biosynthetic enzyme activity and serum LH levels [43]. In contrast, different responses to Flu may result from local concentrations of androgenic and anti-androgenic compounds. Exposure to Flu may block the physiological action of testosterone at AR sites [44] and induce changes in circulating LH levels, due to disturbances in the negative feedback loop between the pituitary and the testis [40]. Although DEHP and Flu share an anti-androgenic activity, the mechanisms by which these EDs exert their effects on human body are distinct. It has indicated in many previous reports that AR plays an important role in Flu-mediated response, while phthalates, including DEHP, do not interact with AR at the physiological concentration [45]. Additionally, DEHP is thought to activate PPAR, leading a down-regulation of SR-B1 and PBR or effects on SF-1 to consequently regulate steroidogenesis-related genes [16]. To further understand the effects of DEHP and Flu during the critical stages of male reproductive development, we measured the serum concentrations of testosterone and LH. However, exposure to all doses of Flu and DEHP failed to induce a significant effect in the serum LH levels, while a significant decreased level in the serum concentrations of testosterone was observed in response to all doses of DEHP. We did not expect to observe Flu-induced effect on serum concentrations of testosterone and LH, however, the response of Flu on their serum concentrations may be explained by the lack of a negative feedback mechanism. Further experiments with larger sample sizes are required to verify these findings. It has been indicated that exposure to a high dose of DEHP caused a decrease in testosterone production and consequently reduced AGD value [46]. In this study, serum testosterone concentration and AGD value were reduced in response to a high dose of DEHP, suggesting that an alteration in testosterone synthesis may result from phthalate-induced dysfunctional interaction between Leydig and Sertoli cells [47]. Thus, the pathological changes induced by anti-androgenic effects in the testis during male reproductive tract development may alter serum testosterone levels.

Testes are androgen-responsive tissues, and alterations in the morphology and histology of testes can reflect the anti-androgenicity effects of chemicals. In addition, an evaluation of histological and biological effects is very important to understand the potential risk impacts for spermatogenesis from endocrine disrupting chemicals [48]. In this study, histopathological changes in the testes of immature male rats exposed to DEHP and Flu were examined, and abnormalities in the cell morphology and histology of the testes were observed (i.e., abnormal testicular development or dysgenesis, Leydig cell hyperplasia, morphologically distorted tubules, differentiated Sertoli cells and abnormal germ cells), particularly in response to high doses of DEHP and Flu. A previous study indicated that in utero exposure to DEHP (1,000 mg/kg BW) caused the dilatation and atrophy of seminiferous tubules in rats. In addition, exposure to DEHP (500 mg/kg BW) may result in the abnormalities of the cell morphology in which multinucleated germ cells were observed in seminiferous cords [49]. Another study has indicated the age-related difference in the toxic effect(s) of this ED in the testis of rats [50]. A testicular atrophy and/or a decline in zinc concentration were observed when exposure of immature rats to DEHP [51], demonstrating the DEHP- and Flu-induced effects on male reproductive development, particularly with regards to spermatogenesis.

A previous study has investigated the alteration in the gene expression following DEHP treatment in which exposure to this compound caused a dysregulation in the expression of many genes, including apoptosis-, cell proliferation-, metabolism-, cell adhesion- and immune response- related genes [52]. In this study, the gene expression in immature rat testes was also assessed via cDNA microarray assays. A total of 37,317 genes were dysregulated in response to DEHP (i.e., 100 mg) and Flu (i.e., 10 mg) treatment, when compared with a control. Among these genes, 1,272 were overexpressed by more than two fold, and 1,969 genes were downregulated (Table 2 and 3). Some of these genes were chosen for use as marker genes. We previously demonstrated that maternal exposure to DEHP and Flu modulates the expression of fetal *StAR, Cyp11a1 *and *Hsd3b1 *genes, wherein DEHP treatment downregulated transcription [53]. However, no significant effects were observed in the current study. Interestingly, treatment with high doses of Flu significantly increased the expression of these genes (i.e., by 4- to 6-fold). Several genes shown in Table 2 and 3 [i.e., calcium binding protein 1(*CaBP1*); vav2 oncogene- predicted (*Vav2- predicted*); phospholipase C delta 1 (*Plcd1*); lim homeobox protein 1 (*Lhx1*) and isochorismatase domain containing 1 (*Isoc1*)] were previously identified because of their direct or indirect involvement in physiological processes (e.g., lipid or steroid metabolism, sex determination, calcium transduction or cell proliferation). However, the biological interactions of these proteins in response to EDs (i.e., particularly DEHP and Flu) remains unknown. In current study, we also found that some genes were strongly up-regulated by TP treatment, whereas no differences at transcriptional levels were observed in the testes of rats exposed to DEHP and/or Flu. Furthermore, expression of other genes, including nuclear receptor interacting protein 3 (predicted) and phosphatidylinositol glycan, class V, were decreased markedly in TP treated group, while an enhancement in expression of these genes was observed following DEHP or Flu treatment. A distinct response to TP and DEHP/Flu may reflect hormone properties of tested chemicals. Additionally, a different pattern of gene expression also was found between DEHP and Flu treatment. These distinct responses to DEHP and Flu may be explained by the differential mechanism of actions of the two anti-androgenic EDs. The discrepancy was observed in the microarray results compared to the previous ones, which appears to be derived from different microarray system employed and different experimental environments among the studies.

It has been reported that calcium binding protein-1 (i.e., encoded by *CaBP1*) functions as a calmodulin-like protein that modulates Ca2+ channel activities [54]. An interaction between Ca2+ channels and *CaBP1 *may regulate the Ca2+-dependent forms of synaptic plasticity by inhibiting Ca2+ influx into neurons [55]. In this study, the expression of *CaBP1 *was significantly up-regulated by TP exposure. Our data revealed that exposure to TP activated the predicted *Vav2 *oncogene. The *Vav2 *gene is involved in many biological processes, including the B- and T-cell receptor signaling pathways, leukocyte transendothelial migration, natural killer cell-mediated cytotoxicity and regulation of the actin cytoskeleton. Some previous studies have demonstrated an appearance of *CaBP1 *and *Vav2 *oncogene genes during spermatogenesis, suggesting their potential roles in the critical window stage of male reproductive system development [56,57]. Phospholipase C delta 1 (*Plcd1*), a ubiquitous enzyme, helps regulate a variety of cellular processes. The *Plcd1 *gene has been detected in the plasma membrane, the cytoplasm [58], and liver mitochondria [59], and the involvement of *Plcd *in cardiac function has also been reported [60]. In addition, the activity of *Plcd1 *is affected by cytoplasmic concentrations of Ca2+ [61]. A previous study also demonstrated that FSH-induced Ca(2+) influx is mediated by the *Plcd1 *signaling pathway in rat Sertoli cells [62]. Moreover, a testosterone-induced increase in intracellular Ca2+ occurs via activation of a plasma membrane receptor associated with the phospholipase C signaling pathway [63]. Our results revealed a significant increase in the expression of its pathway. In addition, we found that the expression of LIM homeobox gene 1 (*Lhx1*) was significantly modulated by DEHP, suggesting that this gene is a potential indicator for the effects of DEHP on androgen-responsive tissues. As a member of the large homeobox gene family, *Lhx1 *plays an important role in the stabilization of the intermediate mesoderm and in the formation of urogenital ridges [64]. This gene is expressed in the comma- and S-shaped bodies of the metanephric mesenchyme, as well as the developing Müllerian duct [65]. In the male reproductive system, *Lhx1 *plays an important role in early gonad development, sex-reversal and gonadal cell proliferation. Several studies have reported the involvement of *Isoc1 *in progressive chronic renal failure in rats [66]. Sexual hormones (e.g., estradiol, testosterone, prolactin and FSH) concentrations are commonly affected by chronic renal failure and the dysfunction of sex steroids may contribute to the emergence of renal osteodystrophy [67]. Our results showed a 6-fold downregulation in *Isco1 *gene expression after exposure to DEHP, representing an anti-androgen-like ED exposure. These results demonstrate the effects of EDs on sexual hormone abnormalities, and are likely to induce similar changes in the reproductive development of male humans and rats. Overall, our data indicated that the these changes may reflect an impact of chemical exposure on transcriptional level of gene expression and associated with histology changes of Leydig cells, Sertoli cells and/or germ cells, however the role(s) of these altered genes to explain a change in altered phenotype or morphology has unknown. It is expected for us to investigate the molecular events of these maker genes in the histopathological testis changes when EDs exposure during the critical male reproductive development stage in a further study.

Taken together, our findings indicate that exposure to DEHP and Flu results in an alteration in gene expression in the testes of immature male rats. In addition, these chemicals exert distinct anti-androgenic effects on the male reproductive system. Our findings provide new insight into the molecular mechanisms underlying the detrimental impacts of anti-androgenic-EDs in androgen-responsive organs, in particular, in developing male reproductive tracts.

## Competing interests

The authors declare that they have no competing interests.

## Authors' contributions

TTPV and EMJ carried out the overall experiments to complete this study and drafted the manuscript. VHD and YMY performed experiments, in part, and drafted the manuscript. KCC performed real-time PCR and drafted and completed the manuscript. FHY participated in its design and coordination and performed the statistical analysis. EBJ conceived of the study, and participated in its design and coordination and helped to draft the manuscript. All authors read and approved the final manuscript.
